# Q-Switched Nd: YAG in the Treatment of Xanthelasma Palpebrarum

**DOI:** 10.4103/0974-2077.69030

**Published:** 2010

**Authors:** Feroze Kaliyadan, AD Dharmaratnam

**Affiliations:** *Department of Dermatology, Amrita Institute of Medical Sciences and Research Centre, Kochi, Kerala, India. E-mail: ferozkal@hotmail.com*

Sir,

Q-switched Nd: YAG lasers have been advocated as one of the potential treatment options for xanthelasma palpebrarum.[1] Recent studies, however, have questioned the efficacy of this treatment modality.[2] We used the same on a patient with bilateral xanthesmata below the lower eyelids, and wish to report our experience. While the results were not dramatic, there was relatively more improvement on the side where more passes were performed per sitting. However, this was accompanied by more prominent postinflammatory changes.

A 57-year-old female patient presented to our outpatient department with bilateral xanthelasma palpebrarum below the lower eyelids. All available options along with their relative advantages and disadvantages were explained in detail to the patient. After taking an informed consent, the patient was treated with three sittings of a 1,064 nm Q-switched Nd:YAG laser, two sittings at 2-week intervals, 60% energy (~5J/cm ^2^), three to four passes per sitting over the right eye and five to six passes per sitting over the left eye. The patient was evaluated 2 weeks after the second sitting. The pre- and postprocedure photographs were compared.

The procedures were performed after occlusion with local anaesthetic cream (EMLA). The patient had no major discomfort or complications during and after the procedure. Two weeks after the second sitting, the lesions were assessed and it was found that while the lesion on the left side showed some improvement (greater than 50% flattening), the lesion on the right side did not show any significant improvement. However, the left side also showed prominent postinflammatory pigmentary change [Figures 1 and 2]. We would like to conclude that any benefit that Q-switched Nd:YAG laser has on xanthelasma may be dependant on the dose and number of passes per sitting. It is, however, obvious that the more the dose/number of passes, the more the risk of pigmentary changes and scarring. Therefore, the mild benefit obtained is offset by the side-effects, thereby limiting the utility of this modality.

**Figure 1:**
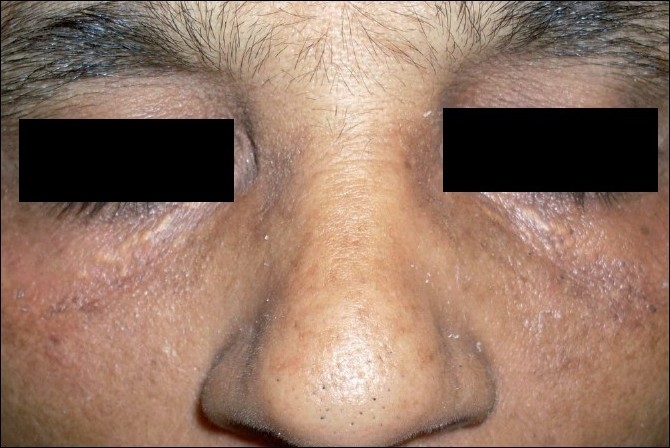
Preprocedure: bilateral xanthelasma below the eyelids

**Figure 2:**
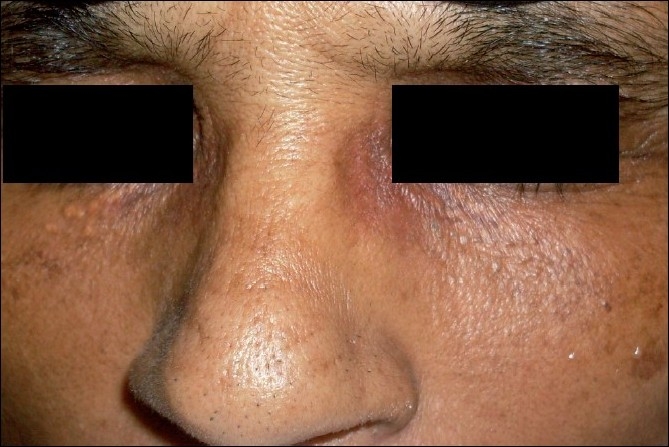
After two sittings of Q-switched ND:YAG laser

Two relatively recent studies have shown conflicting results in the context of using Q-switched Nd:YAG in the treatment of xanthelasma. The study by Fusade[1] was the first major study that demonstrated good results in the treatment of xanthelasma palpebrarum with 1,064 nm Q-switched Nd:YAG laser. However, a subsequent larger study by Karsai *et al*.[2] could not replicate a similar good result. Karsai *et al*. went on to conclude that the treatment of xanthelasma palpebrarum with 1,064 nm Q-switched Nd:YAG LASER cannot be recommended based on the present available evidence.[2]

The perceived advantages of using a Q-switched Nd:YAG laser for xanthelasma palpebrarum would include the avoidance of major side-effects such as atrophy, scars, blisters, hypopigmentation or hyperpigmentation. *In vitro* studies have shown that fatty tissue can be selectively targeted with laser light of 1,064 nm,[3,4] but these results refer to subcutaneous tissue containing predominantly triglycerides.[2]

Therefore, while there appears to be a hypothetical basis for the use of Q-switched Nd:YAG laser for xanthelasma palpebrarum, the clinical benefits have not been demonstrated adequately by published data. We would like to agree with the conclusions made by Karsai *et al*.[2] in suggesting that larger randomized controlled studies with a good design are required to evaluate the efficacy of Q-switched Nd:YAG laser in the treatment of xanthelasma palpebrarum.

## References

[1] Fusade T (2008). Treatment of xanthelasma palpebrarum by 1,064-nm Q-switched Nd:YAG laser: A study of 11 cases. Br J Dermatol.

[2] Karsai S, Schmitt L, Raulin C (2009). Is Q-Switched Neodymium-Doped yttrium aluminium garnet laser an effective approach to treat xanthelasma palpebrarum? Results from a Clinical Study of 76 Cases. Dermatol Surg.

[3] Ichikawa K, Miyasaka M, Tanaka R, Tanino R, Mizukami K, Wakaki M (2005). Histologic evaluation of the pulsed Nd:YAG laser for laser lipolysis. Lasers Surg Med.

[4] Anderson RR, Farinelli W, Laubach H, Manstein D, Yaroslavsky AN, Gubeli J (2006). Selective photothermolysis of lipid-rich tissues: A free electron laser study. Lasers Surg Med.

